# New Chinese record of the genus *Spinonychiurus* (Collembola, Onychiuridae), with the description of a new species

**DOI:** 10.3897/zookeys.439.7789

**Published:** 2014-09-08

**Authors:** Xin Sun, Yu Li

**Affiliations:** 1 Engineering Research Center of Chinese Ministry of Education for Edible and Medicinal Fungi, Jilin Agricultural University, Changchun 130118, China; 2 Key laboratory of Wetland Ecology and Environment, Northeast Institute of Geography and Agroecology, Chinese Academy of Sciences, Changchun 130102, China

**Keywords:** Taxonomy, Thalassaphorurini, tibiotarsi, key

## Abstract

A new collembolan species is described, *Spinonychiurus sinensis* **sp. n.**, which has seven chaetae in the distal row of the tibiotarsi. It is placed in the genus *Spinonychiurus* due to two important characters: the two subsegments on Abd. III sternum and the absence of d0 on the head. This is the first report of the genus *Spinonychiurus* in China. The diagnosis of *Spinonychiurus* is broadened and the key to the world species is provided.

## Introduction

The genus *Spinonychiurus* was established by Weiner (1996) for the Scottish species *Onychiurus edinensis* Bagnall, 1935, and it was assigned to the tribe Onychiurini Börner, 1901 as having compound vesicles on the postantennal organ. Kaprus’ and Tsalan (2009) revised the diagnosis characters of the genus and moved the genus into the tribe Thalassaphorurini Pomorski, 1998 by the structure of the furcal area and the distinct S-chaeta on the antennae. So far, only four species of the genus *Spinonychiurus* are reported throughout the world: *Spinonychiurus edinensis*, *Spinonychiurus epaphius* Kaprus’ & Tsalan, 2009 from Ukraine, *Spinonychiurus subedinensis* (Arbea & Jordana, 1985) from Spain and *Spinonychiurus vandeli* (Cassagnau, 1960) from France.

During our study on specimens collected from Changbai Mountain Range, we found a new species closest to the genus *Spinonychiurus* Weiner, 1996 but having 7 chaetae in the distal row of tibiotarsi (there are 11 in *Spinonychiurus* according to Kaprus’ and Tsalan (2009)). In the present paper, we assign the new species to the genus *Spinonychiurus* with two important characters: two subsegments on Abd. III sternum and the absence of d0 on the head. The description of the new species and the broadened diagnosis of the genus are given below. An updated key to the species of the genus *Spinonychiurus* is provided.

## Material and methods

Specimens were mounted in Marc André II solution, after clearing in lactic acid, and were studied using a Nikon Eclipse 80i microscope. Material is deposited in the Key Laboratory of Wetland Ecology and Environment, Northeast Institute of Geography and Agroecology, Chinese Academy of Sciences, Changchun (NEIGAE).

Labial types are identified after Fjellberg (1999). Labium areas and chaetal nomenclature follow Massoud (1967) and D’Haese (2003). Chaetae on anal valves are recognised after Yoshii (1996). Furcal area is classified after Weiner (1996). Tibiotarsus chaetotaxy formula follows Deharveng (1983), and is expressed as: total number of chaetae (number of chaetae in row C, number of chaetae in row B, number of basal chaetae in rows A+T).

Abbreviations used in descriptions and figures:

Ant. – antennal segments, PAO – postantennal organ, Th. – thoracic segments, Abd. – abdominal segments, pso – pseudocellus, psp – pseudopore, psx – parapseudocelli, psxm – unpaired parapseudocelli, ms – microsensillum, p-chaeta – chaeta of row p, S – S-chaeta, Sp – posterior S-chaeta on Abd. V, AIIIO – sensory organ of antennal segment III.

The pseudocelli and pseudopores formulae are the number of pseudocelli or pseudopores per half-tergum or half-sternum. The S-chaetae formula is the number of S-chaetae per half-tergum or half-sternum from head to Abd. VI.

## Systematics

### Family Onychiuridae Börner, 1913
Tribe Thalassaphorurini Pomorski, 1998

#### Genus Spinonychiurus

Taxon classificationAnimaliaPoduromorphaOnychiuridae

Weiner, 1996

##### Type species.

*Onychiurus edinensis* Bagnall, 1935: 117.

##### Diagnosis.

Postantennal organ oval, with numerous compound vesicles perpendicular to the long axis; clubs of AIIIO smooth, ribbed or granulated; Ant. IV with differentiated S-chaetae; posterior pso on head present; chaeta d0 on head absent; S-chaetae on the body well marked; Abd. V tergum with or without spines; Abd. VI with one or two axial chaetae; anal spines present or absent; distal whorl of tibiotarsal chaetae as 7 or 11; Abd. III sternum divided into two subsegment; furcal rudiment as a finely granulated area with 4 small dental chaetae in two rows posteriorly, one manubrial row of chaetae present posteriorly to dental chaetae.

#### Spinonychiurus sinensis sp. n.

Taxon classificationAnimaliaPoduromorphaOnychiuridae

http://zoobank.org/BC4B8586-C7AE-4DB2-A1FB-E02B17174A8A

Figs 1 –2

##### Type material.

Holotype: female; paratypes: 3 females and 1 male on slides - China, Jilin, Changbai Mountain Range (alt. 689 m, 43.0376°N, 128.9965°E), 3.Oct.2011, litter and soil, Berlese extraction, leg. Tang Xuguang.

##### Diagnosis.

Pso formula as 32/133/33343 dorsally, absent ventrally, subcoxa 1 of legs I-III with 1, 1 and 1 pso respectively; psx formula as 11/000/122211^m^ ventrally, absent dorsally, subcoxa 1 of legs I-III with 1, 1 and 1 psx respectively; S-chaetae formula as 1/011/111021 dorsally, 11/000/000110 ventrally; sterna of Th. I, II, and III with 0+0, 2+2, 2+2 chaetae respectively; Abd. IV tergum with axial chaeta p0, Abd. V tergum with a0 and m0, Abd. VI tergum with a0; the distal row of tibiotarsi with 7 chaetae; male ventral organ absent; anal spines present, 0.8 times as long as inner edge of hind unguis.

##### Description.

Body white in alcohol. Size 970–1200 µm in females, 900 µm in male; holotype: 1200 µm. Body subcylindrical, body sides parallel.

**Figure 1.**
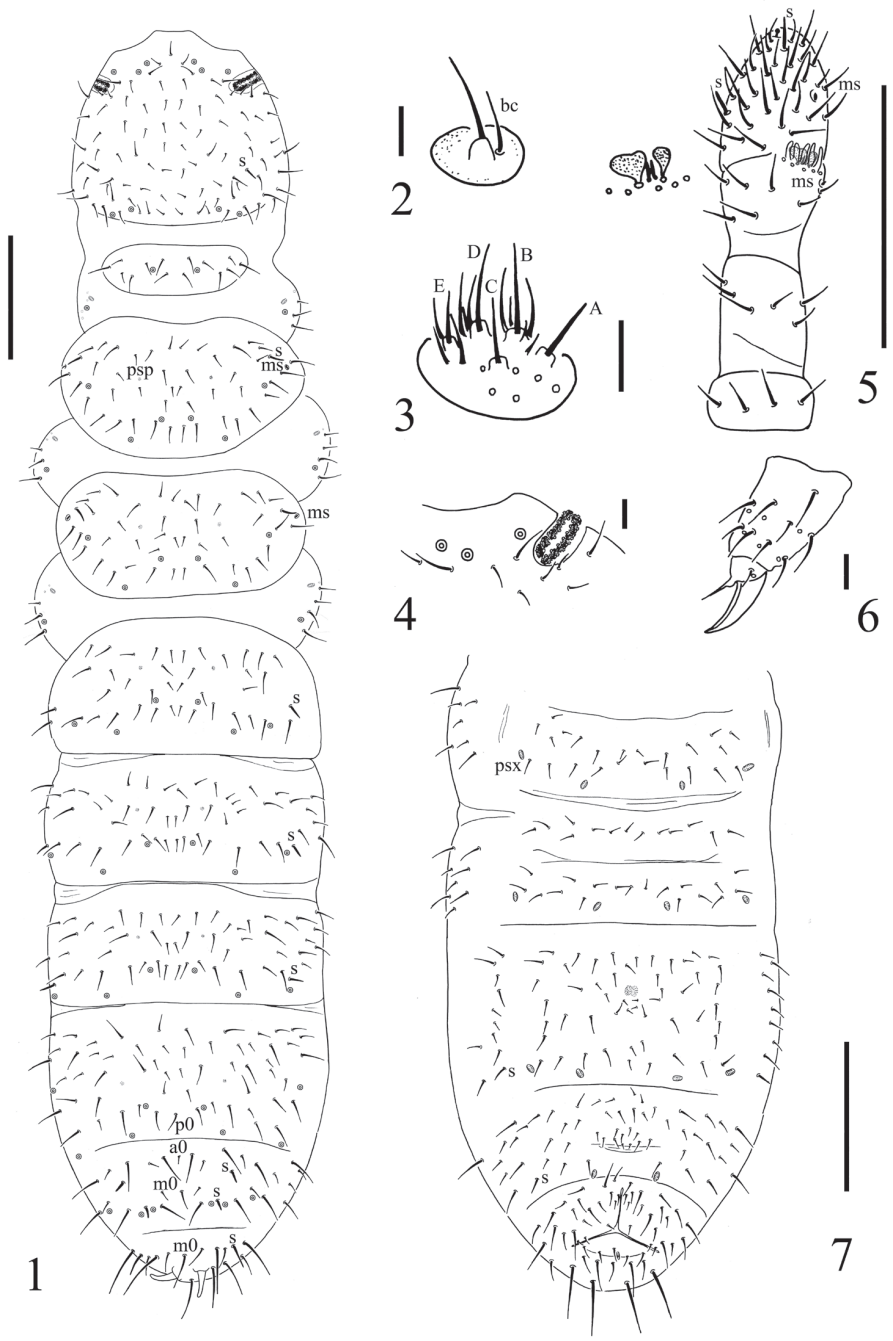
*Spinonychiurus sinensis* sp. n. **A** dorsal chaetotaxy of body **B** maxillary palp **C** labium **D** postantennal organ **E** antenna **F** distal part of leg III **G** chaetotaxy of Abd. II–VI sterna. Scale bars: 0.1 mm (**A, E, G**), 0.01 mm (**B–D, F**).

**Figure 2.**
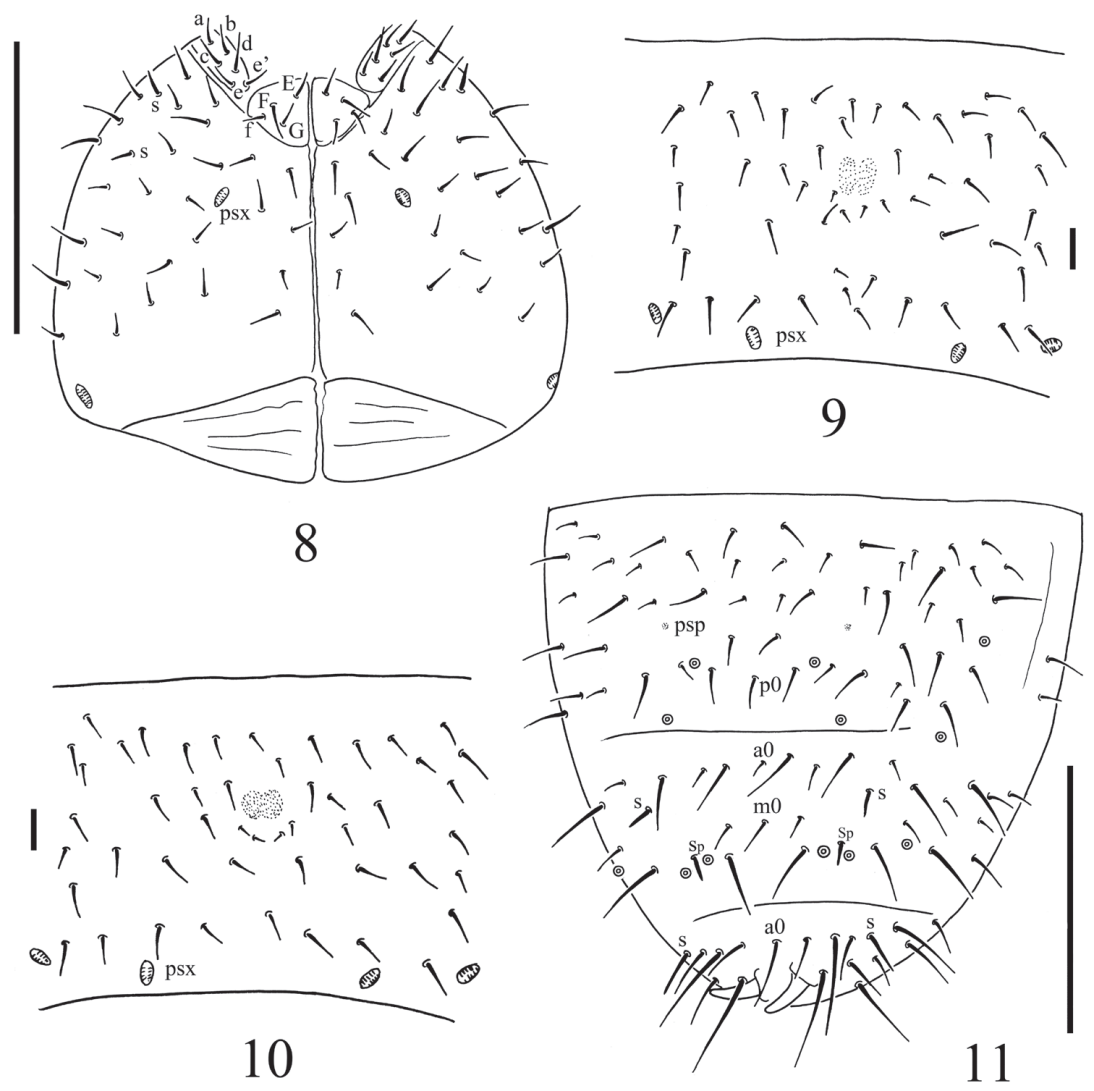
*Spinonychiurus sinensis* sp. n. **A** ventral chaetotaxy of head **B–C** central part of abdominal sternum IV **D** dorsal chaetotaxy of Abd. IV–VI. Scale bars: 0.1 mm (**A, D**), 0.01 mm (**B–C**).

Pseudocellar formula: 32/133/33343 dorsally, absent ventrally (Figs 1A, G, 2A), subcoxa 1 of legs I–III with 1, 1 and 1 pso respectively. Parapseudocellar formula: 11/000/122211^m^ ventrally, absent dorsally (Figs 1A, 1G, 2A), subcoxa 1 of legs I–III with 1, 1 and 1 psx respectively. Pseudopore formula: 0/011/11110 dorsally, -/111/- ventrally (Figs 1A, G, 2A).

Head. Antennae short and distinctly segmented, as long as head. Length ratio of Ant. I: II: III: IV as about 1: 2: 2: 2. Ant. IV with two distinct thickened S-chaetae, subapical organite with globular apex; basolateral ms at approx. half length from base, above the second proximal row of chaetae (Fig. 1E). AIIIO composed of 5 papillae, 5 guard chaetae, 2 sensory rods and 2 smooth sensory clubs, the inner bigger than the outer, and a lateral ms (Fig. 1E). Ant. II with 15 chaetae. Ant. I with 9 chaetae. Antennal base not marked (Fig. 1D). PAO with 12–13 compound vesicles (Fig. 1D). Dorsal cephalic chaeta d0 absent (Fig. 1A). 3+3 p-chaetae present between two inner posterior pso, p1 in line with others (Fig. 1E). Mandible with strong molar fig and 4 apical teeth. Maxilla bearing 3 teeth and 6 lamellae. Maxillary palp simple with 1 basal chaeta, without sublobal hair (Fig. 1B). Labral formula 4/1,4,2; labium with 6 proximal, 4 basomedian (E, F, G, f) and 6 basolateral (a, b, c, d, e, e’) chaetae (Fig. 2A); labial type A, papillae A–E respectively with 1, 4, 0, 3 and 3 guard chaetae (Fig. 1C). Head ventrally with 4+4 postlabial chaetae along ventral groove (Fig. 2A).

Body chaetotaxy. S-chaetae subcylindrical, apically rounded, 1/011/111021 dorsally, 11/000/000110 ventrally (Fig. 1A, G); subcoxae 2 of legs I, II and III with 0, 0, 1 S-chaeta respectively. Tiny and blunt ms, present on Th. II–III (Fig. 1A). Ordinary chaetae differentiated into meso- and macrochaetae, ratio Sp: m1: p1 on Abd. V tergum = 1: 1: 2.5. Th. I tergum with 7+7 dorsal chaetae. Th. II–Abd. III terga with 3+3 chaetae along axis respectively (Fig. 1A). Abd. IV tergum with one axial chaeta (p0), Abd. V tergum with two axial chaetae (a0 and m0), Abd. VI tergum with one axial chaeta (a0) (Figs 1A, 2D). Sterna of Th. I, II, and III with 0+0, 2+2, 2+2 chaetae respectively.

Appendages. Subcoxa 1 of legs I–III with 4, 5 and 5 chaetae, subcoxa 2 with 1, 4 and 4 chaetae respectively. Tibiotarsi of legs I, II and III with 16 (1, 8, 7), 16 (1, 8, 7) and 15 (1, 7, 7) chaetae each (Fig. 1F). Unguis without teeth. Unguiculus approx. 0.6 times as long as inner edge of unguis, with inner basal lamella (Fig. 1F). Ventral tube with 4+4 basal and 7+7 distal chaetae. Furca reduced to a field of fine granulation with 4 small dental chaetae arranged in 2 rows posteriorly; only one manubrial row of chaetae posterior to dental chaetae (Figs 1G, 2B, C).

Genital fig with 9–11 chaetae in females, 32 chaetae in male. Male ventral organ absent. Anal valves with numerous acuminate chaetae; each lateral valve with a0, 2a1, 2a2; upper valves with chaetae a0, 2b1, 2b2, c0, 2c1, 2c2. Anal spines set on distinct papillae, 0.8 times as long as inner edge of hind unguis.

##### Derivatio nominis.

Named for the first recordof the genus *Spinonychiurus* in China.

##### Ecology.

Found in the coniferous forest.

##### Discussion.

The new species is closest to the genus *Spinonychiurus* in two important characters: two subsegments on Abd. III sternum and the absence of d0 on the head. However, it does not fit the current definition of the genus as proposed by Kaprus’ and Tsalan (2009) in having 7 chaetae in the distal row of tibiotarsi instead of 11. The distal tibiotarsal chaetae have been verified to be an unstable character in the generic level (Sun et al. 2010; Sun et al. 2011; Sun and Zhang 2012; Sun et al. 2013), so we propose the placement of the new species in the genus *Spinonychiurus* and broaden its diagnosis accordingly. The main diagnostic characters of all known species of the genus are given in Table 1 and a key to these species is provided below.

**Table 1.** Main diagnostic characters of world species of *Spinonychiurus*.

	*Spinonychiurus sinensis* sp. n.	Spinonychiurus edinensis*	*Spinonychiurus epaphius*	Spinonychiurus subedinensis**	*Spinonychiurus vandeli*
Dorsal pso formula	32/133/33343	32/223/1(?)1(?)3(?)43	5-6,5/4-5,8,8-10/9-13,9-12,9-14,9-14,7-10	34/233/44454	32/233/33343
Ventral pso formula	absent	absent	1/000/00000	1/000/01110	1/000/00000
Number of vesicles on PAO	12–13	14–16	13–16	18–22	22–25
Sensory clubs on AIIIO	smooth	smooth	smooth	smooth	granulated
Chaetae in distal row of tibiotarsi	7	11***	11	11	11***
Male ventral organ	absent	?	present on ventral tube	present on ventral tube	absent
Spines on Abd. V	absent	present	absent	absent	absent
Anal spines	present	present	absent	present	present

* The details of *Spinonychiurus edinensis* follow Bagnall (1935) and Weiner (1996);

** the ventral pso formula has been verified by Javier Arbea (based on the type materials collected in University of Navarra);

*** the number of chaetae in distal row of tibiotarsi in *Spinonychiurus edinensis* and *Spinonychiurus vandeli* has been verified by Louis Deharveng (based on the collections in Muséum National d’Histoire Naturelle).

#### Key to the known species of the genus *Spinonychiurus*

**Table:** 

1	Spines on Abd. V tergum present	*Spinonychiurus edinensis* (Bagnall, 1935)
–	Spines on Abd. V tergum absent	2
2	Chaetae in distal row of tibiotarsi as 7	*Spinonychiurus sinensis* sp. n.
–	Chaetae in distal row of tibiotarsi as 11	3
3	Anal spines absent	*Spinonychiurus epaphius* Kaprus’ & Tsalan, 2009
–	Anal spines present	4
4	Dorsal pso formula as 34/233/44454	*Spinonychiurus subedinensis* (Arbea & Jordana, 1985)
–	Dorsal pso formula as 32/233/33343	*Spinonychiurus vandeli* (Cassagnau, 1960)

## Supplementary Material

XML Treatment for Spinonychiurus

XML Treatment for Spinonychiurus sinensis
